# Significant Structural Alterations and Functional Connectivity Alterations of Cerebellar Gray Matter in Patients With Somatic Symptom Disorder

**DOI:** 10.3389/fnins.2022.816435

**Published:** 2022-03-08

**Authors:** Huai-Bin Liang, Liao Dong, Yangyang Cui, Jing Wu, Wei Tang, Xiaoxia Du, Jian-Ren Liu

**Affiliations:** ^1^Department of Neurology, Shanghai Ninth People’s Hospital, Shanghai Jiao Tong University School of Medicine, Shanghai, China; ^2^Shanghai Key Laboratory of Magnetic Resonance, Department of Physics, School of Physics and Electronic Science, East China Normal University, Shanghai, China; ^3^School of Psychology, Shanghai University of Sport, Shanghai, China; ^4^Clinical Research Center, Shanghai Jiao Tong University School of Medicine, Shanghai, China

**Keywords:** voxel-based morphometry (VBM), functional connectivity (FC), cerebellum, somatic symptom disorder (SSD), gray matter volume (GMV)

## Abstract

**Objective:**

Recent studies have revealed a strong association between the cerebellum and psychiatric disorders. However, the structural changes in the cerebellar regions and functional connectivity (FC) patterns in patients with somatic symptom disorder (SSD) have not been elucidated.

**Methods:**

Thirty-seven patients with SSD (29 drug-naive and 8 medicated patients) and 37 sex- and age-matched healthy controls (HCs) were recruited and underwent resting-state functional magnetic resonance imaging scans. The spatially unbiased infratentorial (SUIT) cerebellar atlas-based voxel-based morphometry was used to investigate the changes in cerebellar regional gray matter (GM). Seed-based FC was further computed to explore the pattern of abnormal FC across the whole brain. Correlations were calculated to investigate the relationship between cerebellar structural (and FC) changes and clinical characteristics.

**Results:**

After controlling for age, sex, total intracranial volume, medication, and mean FD covariates, all patients with SSD had increased mean GM volume (GMV) in the posterior lobules of the cerebellum bilaterally when compared with HCs, specifically, in the bilateral cerebellar crura I and II. Patients with SSD showed significantly stronger FC between the right crura I and II and bilateral precuneus inferior parietal region, and postcentral gyrus, extending to the superior parietal lobe, cingulate gyrus, and the white matter subgyral. In addition to the two clusters, right lingual gyrus was also a surviving cluster with significantly higher FC. Partial correlation analysis revealed that the degree of regional GMV increases in the two significant clusters and the Hamilton Depression Scale (HAMD) score was negatively correlated. Moreover, the FC of right crura I and II with the left parietal lobe and right lingual gyrus were also negatively associated with the HAMD score.

**Conclusions:**

SSD exhibited significant microstructural changes and changes in FC pattern in the posterior cerebellar lobe. These results shed new light on the psychological and neural substrates of SSD and may serve as a potential treatment target for SSD based on the cerebellar area.

## Introduction

Somatic symptom disorder (SSD) is a common neuropsychiatric condition that affects an estimated 5–7% of the general population (Kurlansik and Maffei, 2016). SSD is characterized by an extreme focus on physical symptoms, such as pain or fatigue, which cause severe emotional distress and functional problems. Patients with SSD have high rates of depression and anxiety, poor quality of life, frequent doctor visits, and high medical costs. It has become a major public health concern. Thus, better insight into the pathophysiology of SSD may help to develop better diagnostic and therapeutic approaches for these patients.

The well-known function of the cerebellum is to coordinate movement and maintain balance. The cerebellum has been a neglected structure in neuropsychiatric research. Recent evidence indicates that the cerebellum is affected in individuals with mental illness; however, few studies have focused on the role of the cerebellum in SSD (Phillips et al., 2015). The cerebellum has been acknowledged to have many functions, including emotion regulation, impulse inhibition for decision-making, attention, and working memory (Phillips et al., 2015). Patients with SSD often present with excessive somatic complaints and develop excessive attention and sensitivity to physical discomfort, such as pain (Mobley et al., 2019). These findings raise the question of whether the emotional/cognitive regulatory function of the cerebellum may play a role in the pathophysiology of this disease.

Several factors are thought to contribute to SSD. Genetic and hereditary factors, such as hypersensitivity to pain sensations, dysfunctional cognitions, and the personality trait of negativity, may be involved. Some studies have reported that the cerebellum plays a modulatory role in nociceptive processing (Moulton et al., 2010). Experiments using electrical and/or pharmacological stimulation methods have shown that cerebellar activation affects the processing of nociceptive information. In stimulation of rat cerebellar cortex using electrical stimulation or D, L-homocysteine acid increased visceral nociceptive reflexes (Saab and Willis, 2001). There is increasing evidence that the cerebellum is associated with cognition. Patients with cerebellar disease are often found to have “frontal-like” cognitive impairment (Rapoport et al., 2000). Furthermore, in functional neuroimaging studies of healthy individuals, activation of the cerebellum has been observed in tasks involving learning and word generation (Raichle et al., 1994). Previous studies have also suggested that the cerebellum and other brain regions (occipitotemporal regions and prefrontal structures) are involved in negative affect processing, specifically, the cerebelum_crus1_r, cerebelum_crus2, cerebelum_4_5_r, and cerebelum_6_l (Fernandes et al., 2017). However, the role of structural and functional abnormalities in the cerebellum of patients with SSD has not been fully elucidated.

Over the past decade, neuroimaging methods, such as voxel-based morphometry (VBM), positron emission tomography, and functional magnetic resonance imaging (fMRI), have enabled the investigation of structural differences and neuronal activities between different subpopulations. For example, Pan et al. (2019) found that patients with SSD exhibited an increased global functional connectivity (GFC) in the right inferior temporal gyrus and left superior occipital gyrus and a decreased GFC in the right insula compared with healthy controls (HCs). In contrast, Zhao et al. (2020) demonstrated an enhanced thalamocortical functional connectivity (FC) in first-episode, drug-naive patients with SSD. In terms of structural abnormalities, Yildirim et al. (2012) found significantly smaller pituitary volumes in somatization patients compared with HCs. In contrast, Hakala et al. (2004) found that the caudate nucleus volume in female patients with SSD was bilaterally enlarged compared with healthy volunteers. Nevertheless, little is known about the relationship between SSD and the cerebellum, particularly with regard to the changes in cerebellar structural morphology and FC.

Although much attention has recently focused on the role of the cerebrum in other psychiatric disorders, few studies have specifically addressed the pattern of changes in cerebellar morphometry in SSD, the FC patterns, and the relationship between cerebellar gray matter (GM) changes and clinical symptoms. Alterations in cerebellar GM and FC may help in understanding the neurobiological changes leading to SSD. Diedrichsen (2006) developed a subtle spatially unbiased infratentorial template (SUIT) of the human cerebellum and brainstem, providing an improved voxel-by-voxel normalization for fMRI analysis. Thus, in the current study, we investigated the differences in GM volume (GMV) of the cerebellum using VBM of T1-weighted anatomical images, in combination with the cerebellum template from SUIT (Diedrichsen, 2006) to test the following hypotheses: (1) SSD may exhibit altered GMV in the cerebellum; (2) SSD may display altered FC between cerebellar and whole brain; (3) these GM structural alterations may be associated with clinical measures and psychiatric symptoms, such as disease duration, accompanying anxiety, and depression symptom scores.

## Materials and Methods

### Subjects

Patients were recruited from the neurology outpatient clinic of the Shanghai Ninth People’s Hospital and diagnosed with SSD by experienced neurologists according to the diagnostic criteria of the *Diagnostic and Statistical Manual of Mental Disorders, Fifth Edition* (*DSM-5*) (Edition, 2013). We compared VBM of T1-weighted anatomical images between 37 patients with SSD (19 men and 18 women, including 29 drug-naive patients and 8 medicated patients) and 37 age- and sex-matched HCs (18 males, 19 females). Demographic and clinical characteristics data, including age, sex, and disease duration, were recorded. Patients also completed the Hamilton Anxiety Scale (HAMA), Generalized Anxiety Disorder (GAD-7), Patient Health Questionnaire (PHQ-7), and Hamilton Depression Scale (HAMD) to obtain an accurate assessment of disease status. All participants were right-handed, with normal cognitive function and no history of substance abuse; those with common chronic diseases and other neurological and psychiatric disorders were excluded based on clinical examination and structured interviews. The following exclusion criteria for patients with SSD were applied: (1) history of other major psychiatric disorders, including schizophrenia, depression, anxiety, bipolar disorder, substance abuse, or dependence, although comorbid major depression was not considered an exclusion criterion if the appearance of depressive symptoms occurred after SSD onset; (2) primary neurological disorders, such as dementia or stroke; (3) history of neurodevelopmental disorders or neurogenetic disorders; and (4) any significant white matter (WM) changes, such as infarction or other vascular lesions detected by T2-weighted MRI. Drug-naive patients with SSD were defined as patients having their first contact with the neurology or psychiatric department and who had not been previously exposed to antidepressants, antineuropathic pain, or cognitive behavioral therapy (CBT). Patients treated with antidepressant medication, including tricyclic antidepressants or antineuropathic pain drugs such as pregabalin and gabapentin, and CBT were classified as the medicated SSD group. HCs were recruited *via* a local advertisement and were carefully screened through diagnostic interviews. Further exclusion criteria for HCs were a history of psychiatric illness among first-degree relatives and the presence of current or past major medical or neurological disorders.

### Magnetic Resonance Imaging Acquisition

Structural MRI scan was performed using a 3.0-T Siemens Prisma system equipped with a 64-channel head coil at the Shanghai Key Laboratory of Magnetic Resonance (East China Normal University, Shanghai, China). Subjects were instructed not to move to minimize head movement, close their eyes, and relax during the scan. High-resolution T1-weighted anatomical images were obtained by using a fast-acquisition gradient-echo pulse sequence prepared by 3D magnetization with the following parameters: repetition time = 2,530 ms, echo time = 2.98 ms, inversion time = 1,100 ms, flip angle = 7°, number of slices = 192, sagittal orientation, field of view = 256 × 256 mm^2^, and voxel size = 1 × 1 × 1 mm^3^. As previously reported, the mean frame-wise displacement (FD) was calculated during head movement processing (Li et al., 2020). Subjects with mean FD Jenkinson greater than 0.2 were excluded.

### Structural Cerebellar Analysis Using SUIT

The cerebellum-optimized voxel-based structural analysis was conducted using a toolbox called SUIT (Diedrichsen and Zotow, 2015) implemented in Statistical Parametric Mapping software, version 12 (spm12^1^). The SUIT toolbox provides templates for high-resolution mapping of the human cerebellum and brainstem, preserving the anatomical details of cerebellar structures, and specialized procedures for automatically separating cerebellar structures from the cerebral cortex normalizing accurate cerebellar structures to that template. The infratentorial structures were isolated from surrounding tissue and segmented into GM and WM tissue classes using the built-in suit_isolate_seg function. Image quality checks were performed using MRIcron^2^ on the segmented images showing infratentorial structures. Before normalization, the T1 image of each subject was cropped and masked before being resliced into the SUIT space so that no supratentorial GM could distort the results. Finally, the preprocessed images were smoothed using a 6-mm full-width at half-maximum Gaussian kernel (Kühn et al., 2012). During the whole-brain voxel-wise analysis, GMVs between the SSD and HC groups were reviewed using a two-sample *t* test at an initial cluster-forming voxel threshold of *p* < 0.001. All results were family-wise error (FWE) corrected for multiple comparisons (*p* < 0.05). Age, sex, total intracranial volume (TIV), mean FD, and medication were entered as covariates into a general linear model in SPM12. The intergroup comparison was tested as follows: SSD group > HC group.

### Seed-Based Functional Connectivity Analysis

All surviving GMV clusters were defined as regions of interest (ROIs); 0.01- to 0.1-Hz bandpass filter was performed for the functional preprocessed data in MNI space. Subsequently, the mean time series was extracted for all ROIs using the DPARSF software package. The Pearson correlation coefficient with the time series of each voxel in the whole brain was calculated to obtain the FC correlation maps. The correlation coefficients were converted using a Fisher *r*-to-*z* transformation to improve the normality of the distribution. Age, sex, mean FD, and medication were used as covariates to minimize the possible effects of these variables. The results were visualized with the BrainNet Viewer^3^.

### Correlation Analysis

We also performed correlation and linear regression analyses to investigate the linear relationships between the covariates of clinical characteristics and cerebellar GMVs (and FC). Individual mean GMVs of the two significant clusters, as the ROIs, were extracted using the DAPBI toolbox (Yan et al., 2016) for the analysis above with the clinical data, including PHQ-9, GAD-7, HAMA, HAMD, and disease duration. After controlling for age, sex, and medication as confounding variables, the relationship between psychiatric symptom scales and mean GMV values was tested using a partial correlation analysis. Based on the same method above, we also investigated the correlation between the clinical data and the surviving clusters in the FC analysis.

### Statistical Analysis

The independent-samples *t* test and χ^2^ test were applied to compare the demographic variables of SSD and HC using SPSS software (SPSS 22.0; IBM, Chicago, IL, United States). Voxel-wise comparisons of GMV between groups were performed within the framework of VBM. Regional differences were considered significant only if they survived after correction for multiple comparisons (FWE correction at the cluster level, *p* < 0.05). Age, sex, TIV, mean FD, and medication were entered as covariates of no interest into the SUIT-VBM analysis. Partial correlation statistical analysis was conducted between the clinical characteristics and GMV or FC of significant clusters as variables with age, sex, and medication as covariates, The significance threshold of the correlations was set to *p* < 0.05 (uncorrected for multiple comparisons).

## Results

### Demographic and Clinical Characteristics

The mean age (± SD) was 48.24 ± 13.41 years for all patients with SSD (47.17 ± 13.80 years for drug-naive and 52.13 ± 11.89 years for medicated SSD) and 49.22 ± 13.17 years for HCs. There were 19 men and 18 women in the all-patient group and 18 men and 19 women in the control group. There were no significant differences in age, sex, TIV, and mean FD between patients with SSD and HCs. The median disease duration in all patients with SSD was 24 months. The mean scores on PHQ-9, GAD-7, HAMA, and HAMD in all patients with SSD were 8.50 ± 3.94, 7.48 ± 5.50, 10.87 ± 3.71, and 10.05 ± 4.59, respectively. Except for disease duration, no statistically significant differences were observed between drug-naive and medicated patients with SSD according to age, sex, TIV, mean FD, PHQ-9, GAD-7, HAMA, and HAMD. Full demographics and clinical characteristics of patients with SSD and HCs are shown in Table 1.

**TABLE 1:** Demographic characteristics and clinical scores of the SSD group and control group.

	Controls (*n* = 37)	All SSDs (*n* = 37)	Drug-naïve SSD (*n* = 29)	Medicated SSD (*n* = 8)	P_1_-value	P_2_-value
Female/Male	19/18	18/19	14/15	4/4	*0.816*	*1.00*
Age (years)	49.22 ± 13.17	48.24 ± 13.41	47.17 ± 13.80	52.13 ± 11.89	*0.754*	*0.362*
TIV	1,370 ± 123	1,405 ± 135	1,423 ± 127	1,340 ± 149	*0.246*	*0.125*
Mean FD	0.10 ± 0.05	0.08 ± 0.04	0.08 ± 0.04	0.07 ± 0.03	*0.062*	*0.449*
Disease duration (month)	–	24 [6, 54]	12 [5, 30]	63 [11, 114]	–	*0.038*
PHQ9	–	8.50 ± 3.94	10.00 ± 4.73	9.57 ± 3.69	–	*0.829*
GAD-7	–	7.48 ± 5.50	8.50 ± 3.94	9.00 ± 6.16	–	*0.805*
HAMA	–	10.87 ± 3.71	10.76 ± 3.09	11.17 ± 5.46	–	*0.825*
HAMD	–	10.05 ± 4.59	9.75 ± 3.47	10.83 ± 7.17	–	*0.634*

*Data are presented as numbers, mean ± SD, or median [25%, 75%].*

*TIV, total intracranial volume; Mean FD, mean framewise displacement; PHQ9, Patient Health Questionnaire; GAD-7, Generalized Anxiety Disorder; HAMA, Hamilton Anxiety Scale; HAMD, Hamilton Depression Scale. −, No data.*

*The P_1_-value indicates the comparison between all patients with SSD and controls, and the P_2_-value indicates the comparison between drug-naïve and medicated patients with SSD. All P values are identified in italics.*

### Spatially Unbiased Infratentorial Gray Matter Volume Changes

**FIGURE 1:**
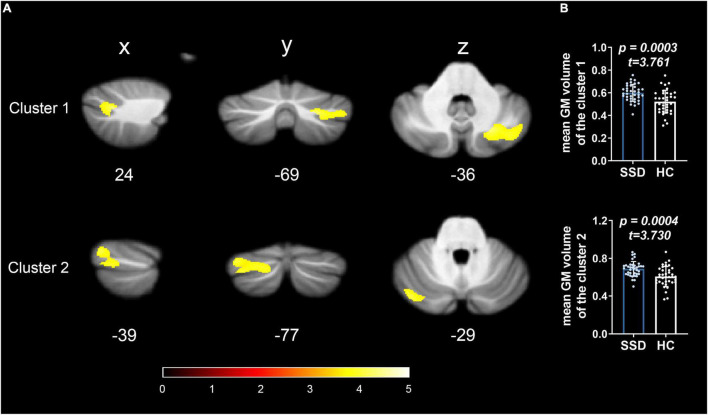
GMV alterations in the cerebellum of patients with SSD detected by spatially unbiased infratentorial–VBM. Compared with HC, patients with SSD showed significantly increased GMV in the two regional clusters in the posterior cerebellar lobes. **(A)** Cluster 1 was located in right lobules crura I and II; cluster 2 was located in the left lobules crus II and crus I. **(B)** The bar graphs show the comparison of the average GMV values, and the error bars represent SD.

**FIGURE 2:**
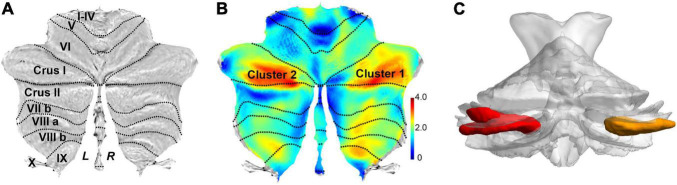
Flat map representation of human cerebellar anatomy and the surviving clusters from spatially unbiased infratentorial (SUIT)–VBM. **(A)** Flat map showed the surface projection of cerebellar anatomy through the SUIT atlas. The posterior cerebellum lobe is anatomically composed of lobules VI, crus I, crus II, VIIb, VIII, and IX. **(B)** Statistical differences in cerebellar GMV between the patients with SSD and HC (SSD > HC) are displayed as cerebellar flat map; significant change in GMV in patients with SSD is displayed in orange. **(C)** 3D presentation of the significance areas: these clusters were used as the ROIs for the FC analysis.

The SUIT toolbox was used to investigate the alterations in GMV of the cerebellum associated with SSD. We found that all patients with SSD showed elevated mean GMV in a cluster comprising right crura I and II compared with HC (*x* = 24, *y* = −69, *z* = −36, *T* = 4.09, 63% and 37% probability, respectively, of belonging to these two specific cerebellar regions, *k* = 1,734 voxels) and a cluster consisting of left crus II and crus I (*x* = −39, *y* = −77, *z* = −29, *T* = 3.80, 54% and 45% probability, respectively, *k* = 2,293 voxels). See Table 2 and Figures 1, 2.

**TABLE 2:** Significant inter-group differences in suitVBM analysis.

Predominant regions in each cluster	Cluster size	Peak T-value	MNI coordinates			Cluster-level *P*-value (FWE-corrected)
			
			x	y	z	
**GMV was higher in SSD patients than in controls**						
**Cluster 1**	1734	4.09	24	−69	−36	*0.040*
		3.69	39	−73	−37	
Right Crus I	1087					
Right Crus II	647					
**Cluster 2**	2293	3.80	−39	−77	−29	*0.024*
			−34	−79	−37	
Left Crus II	1245					
Left Crus I	1040					
Left Crus VIIb	7					

*The results were assigned thresholds at p < 0.001 (voxel-level) and FWE-corrected to p < 0.05 at the cluster level. All P values are identified in italics.*

### Seed-Based Functional Connectivity Differences Between Groups

**FIGURE 3:**
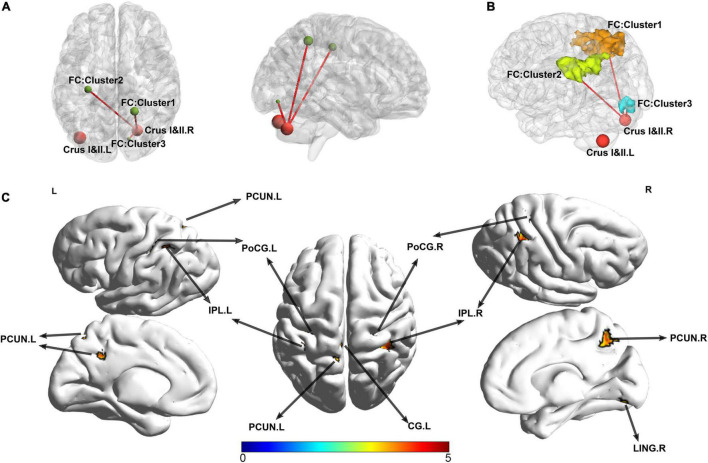
Abnormal functional connectivity patterns in patients with SSD. The surviving clusters in SUIT-VBM were defined as ROIs; FC between the clusters and the whole-brain voxels was calculated in the patients with SSD and HC. **(A)** The significant FCs were presented visually by BrainNet Viewer, sagittal and axial view, respectively. **(B)** The shapes and spatial relationships of all three surviving clusters were outlined in a three-dimensional space. Clusters were labeled with different colors. **(C)** The lateral, medial, and dorsal view of the volume to surface mapping. PCUN, precuneus; IPL, inferior parietal lobule; CG, cingulate gyrus; PoCG, postcentral gyrus; LING, lingual gyrus; SUIT, spatially unbiased infratentorial.

For the cluster located in the right crura I and II regions as the seed point of the ROI, compared with the HC, patients with SSD exhibited increased FC between the right crus I crus II (it is hereafter referred to as ROI 1) and the right parietal lobe, the left parietal lobe, and the right lingual gyrus. The right and left parietal lobes encompass several regions that are similar, such as the precuneus, inferior parietal lobule, superior parietal lobule, postcentral, cingulate gyrus, and a portion of WM called subgyral (Table 3 and Figure 3). Meanwhile, no clusters consistent with statistical significance were found in the FC analysis between ROI 2 and the whole brain.

**TABLE 3:** Significant inter-group differences in ROI-based FC analysis.

Predominant regions in each cluster	Cluster size	Peak T-value	MNI coordinates			Cluster-level *P*-value (FWE-corrected)
			
			x	y	z	
**FC was higher in all SSD patients than in controls (Cluster 1 as ROI)**						
**Cluster 1**	765	5.10	21	−48	51	<*0.001*
		5.03	15	−54	54	
Sub-Gyral	275					
Precuneus	178					
Right Inferior Parietal	98					
Cingulate Gyrus	79					
Right Angular	78					
Right Postcentral	70					
Right Superior Parietal	66					
**Cluster 2**	471	4.84	−30	−24	45	<*0.001*
		4.65	−18	−54	51	
Sub-Gyral	176					
Precuneus	123					
Left Inferior Parietal	118					
Left Superior Parietal	73					
Left Postcentral						
Cingulate Gyrus	46					
**Cluster 3**	71	4.13	15	−78	−9	*0.032*
Right Lingual Gyrus	44					
Right Cerebelum_6	23					
FC was higher in all SSD patients than in controls (Cluster 2 as ROI)						
No clusters survived						

*The surviving clusters were assigned thresholds at p < 0.001 (voxel-level), and FWE-corrected to p < 0.05 at the cluster level. All P values are identified in italics.*

### Correlation With Psychiatric Symptoms

**FIGURE 4:**
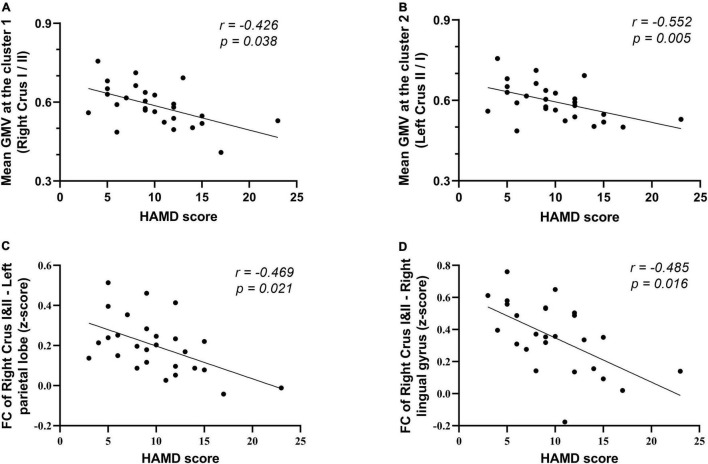
Correlation of altered GMV and FC with the clinical characteristics in the patients with SSD. **(A,B)** Correlation of the mean GMV of two surviving cluster in suit VBM analysis with HAMD score. **(C,D)** Correlation of the FC between the ROI (located on the right crura I and II) and left parietal lobe, right lingual gyrus with HAMD score. Scatterplots for only the significant correlations (*p* < 0.05) are shown here. HAMD, Hamilton Depression Scale.

The clinical characteristics (including disease duration and psychiatric symptom scales) and mean GMV extracted from two significant ROIs were added into the partial correlation analysis after controlling for age, sex, and medication as confounding variables. Altered GMVs of the two clusters showed a significant negative correlation with the HAMD scores (*r* = −0.426, *p* = 0.038; and *r* = −0.552, *p* = 0.005, respectively). No other statistically significant correlations were observed on the disease duration or any other neuropsychiatric scale. As to the significant FC, there is a negative association between the altered FC of ROI 1 and the left parietal lobe region (mainly in the precuneus and inferior parietal, extending to the superior parietal, cingulate gyrus, and the WM subgyral) and HAMD scores; besides, the FC from the clusters of right lingual gyrus also was negatively correlated with the scores (*r* = −0.469, *p* = 0.021; and *r* = −0.485, *p* = 0.016, respectively) (Table 4 and Figure 4).

**TABLE 4:** Correlations between the clinical characteristics and altered GMV (and FC) in the SSD patients.

	Duration	PHQ9	GAD-7	HAMA	HAMD
**Increased GMVs at the cluster 1 (Right Crus I Crus II)**					
r	*0.279*	*0.058*	−*0.129*	−*0.189*	−*0.426*
*p*-value	*0.262*	*0.784*	*0.548*	*0.426*	0.038*
**Increased GMVs at the cluster 2 (Left Crus II Crus I)**					
r	*0.221*	−*0.052*	−*0.168*	−***0****.207*	−*0.552*
*p*-value	*0.378*	*0.804*	*0.432*	*0.382*	0.005**
**Increased FC between ROI 1 and the cluster 1 (mainly located in the right parietal lobe)**					
r	*0.298*	−*0.194*	−*0.085*	−*0.045*	−*0.353*
*p*-value	*0.229*	*0.353*	*0.694*	*0.851*	*0.091*
**Increased FC between ROI 1 and the cluster 2 (mainly located in the left parietal lobe)**					
r	*0.391*	−*0.176*	−*0.112*	−*0.166*	−*0.469*
*p*-value	*0.108*	*0.399*	*0.602*	*0.484*	0.021*
**Increased FC between ROI 1 and the cluster 3 (mainly located in the right lingual gyrus)**					
r	*0.130*	−*0.308*	−*0.130*	−*0.383*	−*0.485*
*p*-value	*0.607*	*0.135*	*0.545*	*0.095*	0.016*

*Partial correlation analysis was conducted under controlling age, sex, and medication as confounding variables.*

*PHQ9, Patient Health Questionnaire; GAD-7, Generalized Anxiety Disorder; HAMA, Hamilton Anxiety Scale; HAMD, Hamilton Depression Scale; r, correlation coefficient.*

*Significant correlations were determined according to p-value < 0.05 (2-tailed, uncorrected for multiple comparisons).*

* indicates p < 0.05, ** indicates p < 0.01. All r and P values are identified in italics.

## Discussion

We examined the localization of cerebellar GM structural differences and FC pattern in patients with SSD and the association between altered cerebellar GMV, FC, and psychiatric symptoms. To our knowledge, this is the first study to investigate the structural differences in the cerebellum in patients with SSD using the SUIT atlas template. We found that the GMV of bilateral crura I and II was consistently increased in patients with SSD compared with HC. We also found consistently enhanced FC between the right crus I/II and the bilateral precuneus and postcentral in patients with SSD compared with HC. Partial correlation analysis revealed the degree of regional GMV increase in the two surviving clusters and showed that the HAMD score was negatively correlated. No statistical correlation was found between disease duration and GMV.

A growing body of research suggests that somatization disorders (SDs) may be associated with certain cognitive styles. There is a broad consensus among researchers and psychologists that patients with SSD form negative cognitive appraisals of their physical sensations and think catastrophically about their feelings. Consistent with this theoretical framework, we found abnormally enhanced GMV in the cerebellar region known as the cognitive cerebellum for the first time. Higher GMV was mainly confined to the posterior cerebellar lobe, anatomically consisting of lobules VI, crus I, crus II, VIIb, VIII, and IX. The cerebellar lobules crura I and II are regarded as anatomical substrates of the cognitive cerebellum (Stoodley and Schmahmann, 2010). The transsynaptic viral tract tracking study confirmed that the prefrontal and posterior parietal cortices are interconnected with crura I and II; the crura I and II were spared anatomical connections to the motor but did show projections to the prefrontal cortex (Eekers et al., 2017). Anterograde transport revealed that prefrontal efferents labeled with herpes simplex virus were found in crus II extending into crus I but were absent in the anterior cerebellar lobe. At the same time, transneuronal retrograde tracing detected that prefrontal afferents were labeled with rabies virus in crus II. Namely, the regions of the posterior cerebellar lobe are functionally coupled to specific cerebral networks. The pattern of GMV changes in the posterior cerebellar lobes of patients with SSD is similar to that in patients with somatic delusional disorder. Higher GMV in the bilateral lobule VIIa/crus II has been detected in patients with somatic-type delusional disorder than in those with non-somatic delusional disorder. Patients with schizophrenia also showed increased GMV in VIIa/crus I compared with HC (Krämer et al., 2020). Task-based fMRI confirmed that crus I is activated by executive functions such as working memory, planning, organizing, and strategy formation. Crus I appears to be involved in encoding generalized aversive processing, exhibiting activation phenomenon in the presence of threatening stimuli (Schmahmann, 2019).

Inconsistent with the present study, investigators also reported a cluster in the right cerebellum crus I, with significantly lower GMV, consisting of 27 voxels (Li et al., 2018). This finding is inconsistent with the results of the present study and may be explained by differences between the study populations as patients with SD included in the previous study did not meet the criteria for SSD (*DSM-5* introduced a new classification category); in addition, in our experience, the detection of small voxel clusters is relatively common when the full-width half-maximum smoothness is limited and often needs to be viewed with caution due to their poor stability. Finally, sex differences in GMV may account for the inconsistency; the proportion of men among SD patients in the study is only 4/25.

The SUIT toolbox offers a high-resolution, spatially unbiased template of the human cerebellum, enabling more accurate intersubject alignment than whole-brain methods (Krämer et al., 2020). In some cases, SUIT is more sensitive to cerebellar changes than conventional whole-brain VBM (Diedrichsen, 2006; Kühn et al., 2012). Increased GMV in the posterior lobules of the cerebellum is observed among patients with SSD. An increase in GMV is often considered as compensatory reallocation or dedifferentiation. Inflammation and astrocyte activation play an important role in this compensatory process, leading to hyperfunction (increased blood flow and metabolism) of the local anatomical regions and increased FC in resting state (Wang et al., 2016). This is supported by the involvement of inflammatory-related molecules in the pathophysiology and treatment response in SSD (Miyauchi et al., 2019; van der Feltz-Cornelis et al., 2020).

Pain symptoms may also reflect underlying structural abnormalities in the cerebellum. Pain is one of the most common symptoms among patients with SSD (Kurlansik and Maffei, 2016; Cozzi et al., 2021; Nazzal et al., 2021). In this study, the most common complaints among patients with SSD were headache, fatigue, and dizziness. The cerebellum is thought to exert a specific role in nociception and somatosensory processing, and many fMRI studies have reported the activation phenomenon in the cerebellum. For example, studies have found that nociceptive trigeminal input is partially processed by the ipsilateral cerebellar lobules crus I, VI, and VIIIa (Mehnert et al., 2017). Increased GMV in the left cerebellar posterior lobe has been found in patients with postherpetic neuralgia (PHN) compared with HC (Liu et al., 2019). Herpes zoster chronification (developing into PHN) can increase GMV and functional changes in the cerebellar posterior lobe (Cao et al., 2018). The underlying process is that central sensitization, chronic pain state, or hyperalgesia induces enhanced glutamatergic signaling, changes in second-order messenger processes, and microglial activation. Thus, human neuroimaging studies have detected anatomical and functional reorganization of the brain level (Baliki and Apkarian, 2015).

Patients with SSD patients showed significantly stronger FC between the right crus I/crus II clusters and bilateral precuneus, inferior parietal lobule, superior parietal lobule, postcentral, and cingulate gyrus. Our findings are consistent with those of previous studies; Li et al. (2018) observed bidirectional corticocerebellar connectivity abnormalities and bidirectional limbic–cerebellar connectivity abnormalities in patients with SD. Previous studies have shown that transcranial magnetic stimulation (TMS) to a lateral region of the cerebellum in crus I/II could increase the FC to the default network such as precuneus, medial prefrontal cortex, and inferior parietal lobule, and the cerebrocerebellar FC also has a causal effect on the cerebral corticocortical network FC (Halko et al., 2014). The precuneus is part of the superior parietal lobule and involved in various complex functions such as memory, emotional response to pain, mental image strategy, recall of episodic memory, and integration of information related to environmental perception. Increased brain activity in the left precuneus is commonly reported in patients with SSD (Kim and Han, 2021). Postcentral is also a part of parietal lobe, which constitutes the somatosensory cortex and plays a role in the integration of somatosensory and has often been shown to be involved in the perception of the painful (Neumann et al., 2021; Tanner et al., 2021). Abnormal FC could affect the integration and processing of the body’s afferent signals, which in turn can lead to physical discomfort. Furthermore, crus I–parietal lobe interactions appear to be relevant in the pathophysiology of neuropsychological disorders (Stoodley et al., 2017). Increased cerebellar default mode network (DMN) connectivity, including the crus I–angular gyrus (inferior parietal lobule) connectivity, and lobule IX–left superior frontal superior medial connectivity has been found in patients with SSD (Wang et al., 2016). The difference is that analysis was based on preassumed seed points with 6-mm radius spheres in the right crus I (MNI: 3, −76, −34) and left crus I (−33, −76, −34) and was focused on the FC with DMN. Similarly, surviving functional connections were not found to be higher in controls than in SSD patients between the seeds and DMN.

Apart from the FC between the right crus I/crus II and the bilateral parietal lobes, we also revealed enhanced FC with the right lingual gyrus. However, the exploration of the role of the lingual in somatic symptoms has been very limited so far. Recent studies have found that the lingual gyrus, in addition to being involved in the encoding of vision, may also mediate the inhibition function and divergent thinking. The GMV of lingual gyrus is positively associated with divergent thinking and inhibition reduction (Zhang et al., 2016). In contrast, it has been previously reported that patients with major depressive disorder (MDD) have reduced right lingual gyrus volume (or density), which is thought to be associated with attention deficits (Couvy-Duchesne et al., 2018). We suspect that the disturbances in attention regulation and inhibition in this region may be involved in the pathological mechanisms, with symptoms manifesting as a heightened preoccupation with somatic feelings.

Based on the positive clusters we found in the cerebellar structural analysis using SUIT and the data we collected, we did not find a statistically significant correlation between disease duration and GMV in the surviving clusters; longer disease duration did not exhibit higher GMV. Accordingly, we hypothesized that the increase in GMV of the posterior cerebellar lobe may contribute to the risk or pathology of SSD. This hypothesis will require corroboration by further research. Furthermore, given that the significant altered GMV regions were calculated after correction for the medication covariate, the positive findings regarding GMV in this study were independent of medication status.

Comorbid psychopathology is common in patients with SSD; the patients often have depressive or anxiety states. Anxiety derives from excessive concern about the symptoms and their potentially catastrophic consequences, and patients may become overtly depressed when symptoms persist and rarely remit for any extended period. Comorbid depression may reflect SSD disease status and suicidality (Wiborg et al., 2013). In the present study, we confirmed that the GMV of surviving clusters was negatively correlated with HAMD scores. This result reflects the fact that higher severity scores of comorbid depressive symptoms were associated with a smaller increase in GMV. Evidence indicates that patients with MDD have reduced GMV in the posterior cerebellar lobes (Peng et al., 2011), including crura I and II, and some treatment choices for depression, such as antidepressants, electroconvulsive therapy, and TMS could partially restore the GMV change (Depping et al., 2017). This finding of a relationship between depression scores and GMV is consistent with previous reports. However, this also implies that when SSD is accompanied by comorbid depression, the degree of GMV increase in these clusters may become less typical.

### Limitation

Our study found that patients with SSD exhibit anatomical changes in the posterior cerebellar lobe; however, several limitations should be noted. First, the sample size was not large, particularly of medicated patients with SSD. Although covariate correction may allow the final findings to exclude the effect of medication, the issue of whether there is a cluster that is affected by medication remains to be addressed. No correlation between disease duration and the clusters was observed in this study, but the effect of disease duration still cannot be absolutely excluded because of the limitations of the sample size and the analysis process. This hypothesis will require corroboration by further research. Second, consistent with most studies in the field, the current study used only HCs and did not use an intervention approach based on drugs or CBT to examine the effects before and after treatment, particularly in the subgroups that responded to treatment. Third, cross-sectional studies are not as valuable as prospective studies for inferring causality. It remains unclear whether the disease status causes structural changes or whether the abnormal cerebellar structure makes the disease more likely to occur. Future research may require a first-episode drug-naive patient cohort study with long-term follow-up to investigate the dynamic changes in cerebellar structure and the corresponding relationship between these changes and treatment response.

## Conclusion

In the present study, we confirmed the presence of posterior cerebellar lobe deficits in patients with SSD, highlighting the role of the crura I and II in disease development. In addition, our data suggest abnormal FC between the traditional frontoparietal cognitive regions and the surviving cluster, which would help us better understand the underlying pathophysiological mechanisms. Future multimodal neuroimaging studies are required to consider the corresponding functional consequences of cerebellar GMV changes concerning the corticocerebellar structural and functional activity in the onset and evolution of the disease.

## Data Availability Statement

The original contributions presented in the study are included in the article/supplementary material, further inquiries can be directed to the corresponding author/s.

## Ethics Statement

The studies involving human participants were reviewed and approved by The Independent Ethics Committee of Shanghai Ninth People’s Hospital. The patients/participants provided their written informed consent to participate in this study.

## Author Contributions

J-RL and XD conceived the project, revised, and edited the manuscript. H-BL and JW were involved in the recruitment and assessment of the subjects. WT performed the MRI scans. H-BL and YC performed the experiments, analyzed the data, and drafted the manuscript and figures. LD helped to analyze and interpret the data. All authors read and approved the final manuscript.

## Conflict of Interest

The authors declare that the research was conducted in the absence of any commercial or financial relationships that could be construed as a potential conflict of interest.

## Publisher’s Note

All claims expressed in this article are solely those of the authors and do not necessarily represent those of their affiliated organizations, or those of the publisher, the editors and the reviewers. Any product that may be evaluated in this article, or claim that may be made by its manufacturer, is not guaranteed or endorsed by the publisher.

## Footnotes

1
http://www.fil.ion.ucl.ac.uk/spm

2
https://www.nitrc.org/projects/mricron

3
www.nitrc.org/projects/bnv/

## Abbreviations

DMN: default mode network
DSM: *Diagnostic and Statistical Manual of Mental Disorders*
FC: functional connectivity
fMRI: functional magnetic resonance imaging
FWE: family-wise error
GAD: generalized anxiety disorder
GM: gray matter
GMV: gray matter volume
HAMA: Hamilton Anxiety Scale
HAMD: Hamilton Depression Scale
HC: healthy controls
ICD: International Classification of Diseases
MDD: major depressive disorder
MNI: Montreal Neurological Institute
PHN: postherpetic neuralgia
PHQ: Patient Health Questionnaire
SD: somatization disorder
SSD: somatic symptom disorder
SUIT: spatially unbiased infra-tentorial
TIV: total intracranial volume
TMS: transcranial magnetic stimulation
VBM: voxel-based morphometry
WM: white matter.
